# Preoperative optimization of cardiac valve patients’ expectations: Study protocol of the randomized controlled ValvEx-trial

**DOI:** 10.3389/fcvm.2023.1105507

**Published:** 2023-03-02

**Authors:** Nicole Horn, Laura Gärtner, Ardawan J. Rastan, Térezia B. Andrási, Juliane Lenz, Andreas Böning, Miriam Salzmann-Djufri, Ulrike Puvogel, Maria Genovese, Sibel Kus, Winfried Rief, Stefan Salzmann

**Affiliations:** ^1^Division of Clinical Psychology and Psychotherapy, Philipps University of Marburg, Marburg, Germany; ^2^Department of Cardiovascular Surgery, Heart Center, Philipps University of Marburg, Marburg, Germany; ^3^Department of Cardiovascular Surgery, Justus Liebig University, Giessen, Germany

**Keywords:** expectations, preoperative psychological intervention, heart valve surgery, clinical trials, placebo

## Abstract

**Introduction:**

Complete recovery after surgery depends on psychological factors such as preoperative information, expectations, and surgery-associated anxiety. Prior studies have shown that even a short preoperative psychological intervention aiming at optimized expectations (EXPECT) can improve postoperative outcomes in coronary artery bypass patients. However, this intervention may benefit only a small subgroup of heart surgery patients since implementing preoperative psychological interventions into the daily clinical routine is difficult due to the additional time and appointments. It is unclear whether the EXPECT intervention can be shortened and whether heart valve patients would also benefit from interventions that optimize patients’ expectations. The multicenter ValvEx trial aims (i) to adapt an effective preoperative psychological intervention (EXPECT) to make it brief enough to be easily integrated into the preoperative routine of heart valve patients and (ii) to examine if the adapted preoperative psychological intervention improves the subjectively perceived illness-related disability (PDI) up to 3 months after surgery.

**Materials and analysis:**

In two German university hospitals, *N* = 88 heart valve patients who undergo heart surgery are randomized into two groups [standard of care (SOC) vs. standard of care plus interventional expectation manipulation (SOC and EXPECT)] after baseline assessment. Patients in the EXPECT group additionally to standard of care participate in the preoperative psychological intervention (30–40 min), focusing on optimizing expectations and have two booster-telephone calls (4 and 8 weeks after the surgery, approx. 15 min). Both groups have assessments again on the evening before the surgery, 4 to 6 days, and 3 months after the surgery.

**Discussion:**

The trial demonstrates excellent feasibility in the clinical routine and a high interest by the patients.

**Ethics and dissemination:**

The Ethics Committees of the Department of Medicine of the Philipps University of Marburg and the Department of Medicine of the University of Giessen approved the study protocol. Study results will be published in peer-reviewed journals and presented at congresses.

**Clinical trial registration:**

ClinicalTrials.gov, identifier NCT04502121.

## Introduction

Heart disease accounts for nearly 10% of all recorded diagnoses in Germany, with an upward trend (1, 2). Cardiovascular diseases are prevalent globally, with a point prevalence of 523 million cases recorded in 2019, and are the leading cause of death worldwide (1, 3). The number of cardiovascular surgeries is also steadily increasing: Between 2008 and 2018, there has been an increase of 23% (4). In 2019, 36 650 heart valve surgeries were performed in Germany (2). The aim of surgical heart valve intervention, such as aortic or mitral valve surgery, is to recover the function of the heart valve through reconstruction or replacement (5). Valve surgeries significantly improve the odds of survival for affected patients (5, 6). However, despite high survival rates after cardiac surgery, the postoperative recovery is highly variable and often unfavorable: a substantial number of patients still feel restricted after surgery, still experience a high illness-related disability, do not perceive an improvement in quality of life, suffer pain or have increased depression scores that persist or even rise for weeks and months after hospital discharge (6–14).

Besides the surgical trauma itself and physical factors like age or general health condition, preoperative psychological factors also influence the recovery process after cardiac surgery (6). Preoperative expectations, illness beliefs (e.g., preoperative illness and treatment-related beliefs), anxiety, and depressive symptoms influence the recovery process, chances of survival, perceived physical and psychological disability, perceived quality of life, and depressive symptoms after cardiac surgery (13, 15–21). Negative expectations before coronary surgery, such as expectations regarding the outcome, the consequences, and the success of treatment, lead to more complications, worse quality of life, higher illness-related disability, increased depressive symptoms, and a postponed return to work (13, 22–24). Given the impact of expectations and illness beliefs on postoperative outcomes and the possibility that these constructs are specifically modifiable through psychological preparation, there is a need to develop preoperative interventions to improve long-term recovery (12, 25).

In the context of the PSY-HEART trial, Rief and colleagues addressed this issue and developed a preoperative intervention (EXPECT) to optimize the patients’ expectations before undergoing coronary artery bypass graft (CABG) surgery (21, 26).

Medical treatments combine both specific factors (e.g., active pharmacological ingredient of a drug or a surgical procedure) and unspecific factors (e.g., patients’ expectations of the treatment outcome), while the unspecific factors of treatments are considered crucial for placebo effects. The EXPECT intervention was based on expectations as one of the most important placebo mechanisms driving placebo effects (27). Placebo effects not only influence subjective dimensions (e.g., quality of life) but have been shown to contribute substantially to surgery outcomes and immune parameters (28). The PSY-HEART-trial indicated effects of the intervention on expectations and depressive symptoms: Patients in the intervention group developed higher personal control expectations, more realistic expectations of the duration of disease, and, in some cases, more positive disability expectations compared with standard of care (SOC) (29). The EXPECT intervention consisted of five appointments: two in-person sessions (approx. 40–60 min) and two telephone calls (approx. 15–20 min) 3–10 days before surgery and one booster telephone call 6 weeks after CABG surgery (30). The positive-realistic expectations developed in the intervention were associated with a better course of treatment 6 months after surgery: The results showed a lower illness-related disability [Cohen’s *d* = 0.75; interpretation of the effect sizes: Cohen’s *d*_small effect size_ = 0.2, Cohen’s *d*_medium effect size_ = 0.5, Cohen’s *d*_large effect size_ = 0.8 (31)], a shorter hospital stay (Cohen’s *d* = 0.46), an earlier return to work (Cohen’s *d* = 0.42), and a higher quality of life (Cohen’s *d* = 0.50) (21, 32). The intervention also influenced physiological parameters such as stress/adrenaline (Cohen’s *d* = 0.34) and immunological parameters (Cohen’s *d*_interleukin–8_ = 0.47, Cohen’s *d*_interleukin–6_ = 0.37) positively (21, 33, 34). However, in the PSY-HEART I trial, almost half of the eligible patients (*n* = 99, 44%) declined to participate in the trial because of difficulties traveling to the additional study appointments, additional time required (four measurement time points and if applicable, approx. 140 min for the intervention), or lack of interest. The additional prehospital intervention appointments and time investment hindered patients’ participation in the trial.

A shortened preoperative psychological intervention at hospital admission could offer more patients accessibility to a preoperative psychological intervention (e.g., by reducing the number of appointments before hospital admission and the associated traveling issues). Another advantage could be that a shorter preoperative psychological intervention would fit better into the current daily hospital routine.

Patients undergoing CABG surgery and patients undergoing heart valve surgery do not differ in the recovery process concerning their anxiety and depression scores (35). Heart valve patients benefit from psychological support (35). This raises the question of whether heart valve patients would also benefit from a preoperative psychological intervention to optimize their expectations. Previous research indicates that heart valve patients would like to receive more information about the psychosocial aspects of their disease and the benefits and risks of treatment options (36). Especially because measures such as health-related quality of life are continuously gaining more relevance, a preoperative psychological intervention enhancing the post-interventional quality of life seems to be a promising approach for patients undergoing heart valve surgery, too (37).

Following the encouraging results of the PSY-HEART I trial (which focused on CABG patients), the ValvEx trial (“Preoperative optimization of cardiac **val**ve patients’ **ex**pectations”) now investigates the efficacy of the EXPECT intervention in patients with planned heart valve surgery compared to a control group (Standard of Care, SOC). To address the problems of the EXPECT intervention in the PSY-HEART I trial (total intervention length and amount of additional contacts), the intervention was modified. It was shortened to make it more suitable for the clinical routine and to minimize the additional effort for the patients (no additional appointments and journeys, less time request; total intervention duration of the EXPECT intervention in the PSY-HEART trial: approx. 140 min; total intervention duration of the EXPECT intervention in the ValvEx trial: approx. 70 min).

If this trial can demonstrate that the expectation intervention (EXPECT) is helpful for heart valve patients, the long-term goal is to implement an interventional treatment protocol for heart valve patients to optimize their expectations in clinical routine care.

## Materials and analysis

### Registration and funding

The trial has been pre-registered at http://www.clinicaltrials.gov (NCT04502121). It is funded by the German Heart Foundation (“Deutsche Stiftung für Herzforschung”) (F/41/20 PI Dr. Salzmann). Table 1 contains all items of the World Health Organization Trial Registration Data Set.

**TABLE 1:** World Health Organization trial registration data set.

Items	Information
Primary registry and trial identifying number	ClinicalTrials.gov, NCT04502121.
Date of registration in primary registry	August 6, 2020.
Secondary identifying numbers	F/41/20 (German Heart Foundation).
Source(s) of monetary or material support	German Heart Foundation (“Deutsche Stiftung für Herzforschung”).
Primary sponsor	Division of Clinical Psychology and Psychotherapy, Philipps University of Marburg, Marburg, Germany.
Secondary sponsor(s)	Department for Cardiovascular Surgery, Heart Center, Philipps University of Marburg, Marburg, Germany; Department for Cardiovascular Surgery, Justus-Liebig-University, Giessen, Germany. German Heart Foundation (“Deutsche Stiftung für Herzforschung”).
Contact for public queries	Nicole Horn, Philipps University of Marburg, Division of Clinical Psychology and Psychotherapy, Gutenbergstraße 18, 35032 Marburg, Germany; telephone: 0049 6421 2823341; fax: 0049 6421 2828904; e-mail address: nicole.horn@staff.uni-marburg.de.
Contact for scientific queries	Stefan Salzmann, PhD. Philipps University of Marburg, Division of Clinical Psychology and Psychotherapy, Gutenbergstraße 18, 35032 Marburg, Germany; telephone: 0049 6421 2823350; fax: 0049 6421 2828904; e-mail address: stefan.salzmann@staff.uni-marburg.de.
Public title	Optimization of patient expectations to improve outcomes in patients undergoing heart valve surgery.
Scientific title	Preoperative Optimization of Cardiac Valve Patients’ Expectations (ValvEx).
Countries of recruitment	Germany.
Health condition(s) or problem(s) studied	Heart valve patients.
Intervention(s)	Intervention name: EXPECT. Intervention description: preoperative psychological intervention with the aim of optimizing expectations.
	Control group: Standard of care (SOC), no treatment.
Key inclusion and exclusion criteria	Ages eligible for study: ≥ 18 years. Sexes eligible for study: both. Accepts healthy volunteers: no.
	Inclusion criteria: scheduled surgery of aortic valve and/or mitral-valve (minimally invasive or open heart surgery).
	Exclusion criteria: emergency surgery, transcatheter aortic valve implantation (TAVI) procedure.
Study type	Interventional.
	Method of allocation: randomized.
	Masking: medical staff.
	Assignment: two-arm.
	Randomization: balanced block randomization (8:8); prestratifications: study center (Marburg or Giessen); and scheduled type of surgery (minimally invasive or open heart surgery).
	Primary purpose: improvement of the recovery process afer heart valve surgery.
Date of first enrolment	June 2020.
Target sample size	88.
Recruitment status	Recruiting.
Primary outcome(s)	Illness-related disability 3 months after surgery.
Key secondary outcomes	Health-related quality of life, expectations, illness beliefs, anxiety, depression, rehospitalization, length of hospital stay.
Ethics review	Status: Approved.
	1. Ethics Committee of the Department of Medicine of the Philipps University of Marburg (AZ 46/20; 2020-05-20).
	2. Ethics Committee of the Department of Medicine of the University of Giessen (AZ 131/20; 2020-08-17).
Completion date	Remaining.
Summary results	Remaining.
IPD sharing statement	Data can be requested on demand.

World Health Organization trial registration data set version 1.3.1.

### Study design

**FIGURE 1:**
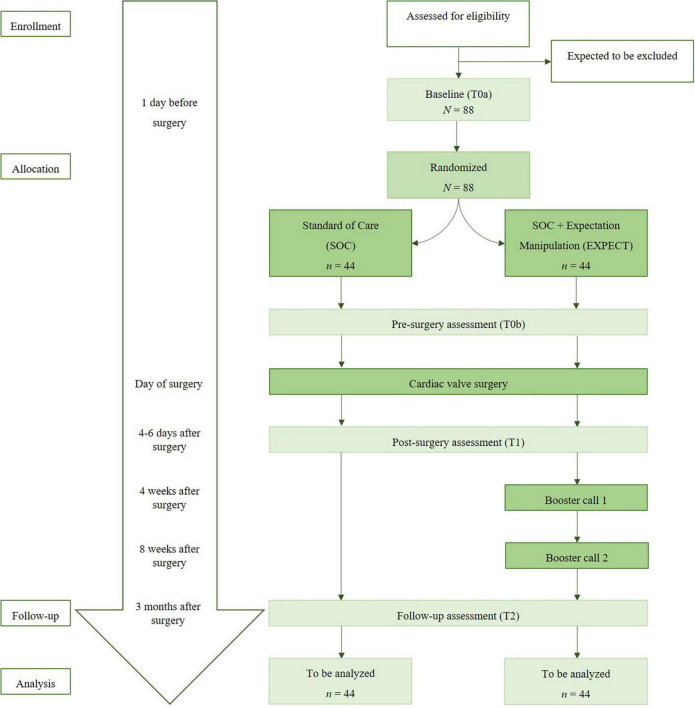
Study flow chart.

The ValvEx-trial is a randomized controlled trial (RCT). It is performed in two centers (the university hospital of Gießen and the university hospital of Marburg). The trial examines the impact of a brief preoperative psychological intervention that aims to optimize expectations (EXPECT) in addition to standard of care compared to standard of care (SOC) only in heart valve patients. The randomization ratio is balanced (1:1). Assessments take place on the day before surgery in the hospital (Baseline, T0a), on the evening before surgery, after the intervention (EXPECT)/after waiting time in the evening of hospital admission (SOC) (T0b), 4 to 6 days after surgery (T1) and 3 months after surgery (T2). Figure 1 shows the calculated study flow-chart, including the participants’ targeted count for each measurement.

### Sample size and study power

In PSY-HEART I, a large effect (*d* = 0.8) was detected relating to the pre-post comparison of the primary outcome (illness-related disability) in the EXPECT and SOC group (21). Since the intervention is shortened to one preoperative session (and two additional booster sessions after surgery) in ValvEx, a small to medium effect is expected (*d* = 0.2–0.5). Given a power (1 − β) of 0.8 and a significance level of *p* = 0.05, a sample size of *N* = 74 is required to detect the described effect (*d* = 0.3, *f* = 0.15). In due consideration of a drop-out rate amounting to 20% for refusals and terminations of treatment, a recruitment aim of *N* = 88 is intended.

### Recruitment and enrollment

Recruitment began at the end of July 2020. Due to the COVID-19 pandemic, elective surgeries were suspended in the meantime, complicating recruitment, which is currently ongoing. The anticipated termination of the trial is at the end of March 2023. Since the trial begins only upon admission to the hospital and no intervention appointments are scheduled before that time, no changes in implementation were necessary during the COVID-19 pandemic. Cardiac valve patients on waiting lists in heart surgery centers are screened. To generate generalizable outcomes and consider the internal validity simultaneously, the following in- and exclusion criteria were created:

Inclusion criteria are:

• Scheduled surgery of aortic valve and/or mitral valve (minimally invasive or open heart surgery),
• Age ≥ 18 years,
• Fluency in German and
• Capability to informed consent.

Exclusion criteria are:

• Comorbid medical/psychiatric condition that causes more extensive disability than the coronary condition,
• Participation in other studies, e.g., PSY-HEART II; in agreement with the coordinating investigator, a patient can participate in cardiovascular studies if this does not interfere with the main study,
• Emergency surgery and
• Transcatheter aortic valve implantation (TAVI) procedure.

Cardiac valve patients on waiting lists of the heart surgery centers in Marburg and Gießen who fulfill the inclusion criteria receive an information brochure about the study with their standard letter confirming the surgery date via post. Patients interested in participating in the study contact the study coordinators by phone or by a response letter. In a first telephone call, the study coordinators answer questions and schedule the study participation for interested patients on the day of their hospital admission. The patient information is sent home to interested patients so they can read it at their own pace. On the day of admission, a physician or a psychologist talk about the patient information with each patient, and patients will have an opportunity to ask questions about the study before providing verbal and written consent to the study.

### Assessment

Patients answer questionnaires at four points of time: at baseline on the day of admission (T0a, approx. 20–25 min), after the intervention (EXPECT)/after waiting time in the evening of hospital admission (SOC) (T0b, approx. 5–10 min), 4 to 6 days after surgery (T1, approx. 10–15 min) and 3 months after surgery (T2, approx. 10–15 min). Table 2 shows the applied questionnaires, case report forms, and biological parameters.

**TABLE 2:** Overview of assessment.

Measurement	T0a: Baseline	T0b: Pre-surgery	T1: Post-surgery	T2: Follow-up
**Questionnaires**				
Illness-related disability (PDI)	X		X	X
Health-related quality of life (MLHFQ)	X		X	X
Expected illness-related disability (PDI-E)	X	X	X	X
Quality of life (SF-12)	X		X	X
Illness beliefs (B-IPQ)	X	X	X	X
Expected illness beliefs (IPQ-E)	X		X	X
Situational cognitive valuations (PASA)	X	X	X	
Preoperative anxiety level and information requirement (APAIS)	X	X		
Anxiety (GAD-7)	X		X	X
Depression (PHQ-9)	X		X	X
Dispositional optimism (LOT-R)	X		X	X
Personality (BFI-10)	X			
Self-stigma	X			
Experience with previous surgeries	X			
Demographics (e.g., age, gender)	X			
Relationship	X			
Interest in/Satisfaction with the intervention	X	X		X
Complications				X
Rehabilitation				X
Conversations with other patients				X
**Case report form (CRF)**				
Blood pressure	X		X	
Planned surgical procedure	X			
Realized surgical procedure			X	
Previous surgeries	X			
BMI	X			
Left ventricular ejection fraction (LVEF)	X		X	
NYHA score	X		X	
EuroSCORE II	X		X	
STS-score	X		X	
Smoking	X			
Clinical data (e.g., duration of surgery, hospital stay, Intensive Care Unit)			X	
Complications			X	
**Biological parameters**				
Inflammatory processes (CRP)	X		X	
Platelets and leukocytes	X		X	

PDI, Pain Disability Index (38); MLHFQ, Minnesota Living with Heart Failure Questionnaire (39, 40); PDI-E, Pain disability index-expectation (26); SF-12, short-form health survey (41); B-IPQ, brief-illness-perception questionnaire (42); IPQ-E, expected illness-perception questionnaire (IPQ-R) (26); PASA, primary appraisal, secondary appraisal (43); APAIS, amsterdam preoperative anxiety and information scale (44, 45); GAD-7, generalized anxiety disorder scale (46); PHQ-9, patient health questionnaire (47); LOT-R, revised life orientation test (48, 49); BFI-10, big five inventory (50); Self-stigma (51); NYHA, New York Heart Association Class (52); EuroSCORE II, European System for Cardiac Operative Risk Evaluation (53); STS-score, society-of-thoracic-surgeons-score (54, 55).

Questionnaires completed during the time in the hospital are personally handed out to the patients by the study staff. The T2 questionnaire is sent home to the study participants. Here, patients will be asked to complete the questionnaire and to contact study staff if they have any questions. To increase responses to all questions and avoid missings, patients receive an explanation letter on how to complete the questionnaire along with the T2 questionnaire. The completed questionnaire can be sent back to the study team in a return envelope. If patients have not returned questionnaires after 2 to 4 weeks, they will be contacted by telephone to clarify possible questions and asked to respond.

### Random allocation

After baseline assessment, the patients are randomized to the intervention group (EXPECT) or the control group (SOC). Therefore, the interventionist opens a prepared, concealed envelope to allocate a patient. The randomization result was prepared in this envelope by a student assistant who was not involved in the study. The interventionists and the patients do not know the randomization result before the envelope is unclosed. Randomization was done as block randomization with a balanced allocation ratio (1:1) using WINPEPI (56). Two prestratifications were considered: The study center (Marburg or Giessen) and the scheduled type of surgery (minimally invasive or open heart surgery).

The interventionists will inform the patients about the group they are randomly assigned to. The medical staff will not receive any information about the group a patient is allocated to and therefore be blind regarding group assignments. The intervention group receives a preoperative psychological intervention to optimize expectations and standard of care. The control group receives the standard of care only.

### Psychological intervention

**FIGURE 2:**
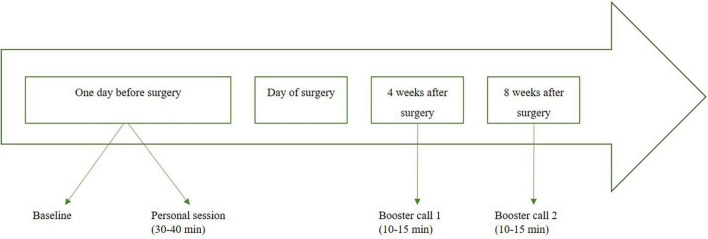
Intervention schedule for EXPECT [adapted from Laferton et al. (26), Salzmann et al. (57)].

For an overview of the implementation of the intervention, please see the intervention schedule (Figure 2). By default, the intervention (EXPECT) takes place on the day of hospital admission. It lasts approximately 30–40 min. Additionally, two booster calls are implemented 4 and 8 weeks after surgery (10–15 min). In case of short-dated deferrals of surgeries for more than 14 days, one additional booster call is implemented.

The EXPECT intervention is performed by one of four trained psychology students shortly before graduating based on an intervention manual. The same person conducts the entire intervention of one patient. The intervention sessions are videotaped and supervised for verification of manual fidelity. Each patient in the intervention group receives a booklet with information material and worksheets to remember discussed contents.

Content and procedure are based on the EXPECT intervention of the PSY-HEART I-trial [for more details, please see Rief et al. (21), Laferton et al. (26), Salzmann et al. (30)]. The intervention deals with optimizing individual expectations relating to the surgery and the recovery process since the optimization of positive, realistic expectations as part of placebo effects is expected to improve the recovery process in patients undergoing valvular surgeries. Since expectations have been shown to be a central characteristic of depressive and anxiety symptoms and mental disorders (27), the development and amplification of positive-realistic expectations as well as the correction of dysfunctional expectations and illness beliefs within the intervention should also influence psychological factors such as levels of anxiety or depressive symptoms.

Based on the integrative model of expectations in patients undergoing medical treatment (58), generalized self-efficacy, treatment outcome expectations (benefit expectations), timeline expectations, and personalized outcome expectancy are addressed in the EXPECT intervention. Optimization of expectancy effects refers to two main objectives: First, to optimize positive expectations regarding treatment success and impairment after medical heart valve surgery. Second, the intervention should specifically promote positive expectations about one’s coping skills in case of ordinary but unpleasant concomitant effects and potential aversive events due to the surgical procedure. By optimizing the expectations before surgery, the treatment success and recovery process should be improved.

While performing the intervention, the personal disease model and experienced impairments are first inquired. If necessary, the interventionists supplement or correct the disease model. After that, a list of activities is used for a discussion illustrating which activity can be done at which timepoint after surgery (e.g., go for a walk: in the first 6 weeks after surgery, lawnmowing: 6 weeks after surgery, skydiving: 3 months after surgery). Interventionists and patients identify which activities are some the patient looks forward to in order to optimize the patient’s benefit and timeline expectations. Next, patients are educated about risk factors and health behavior before an individual health plan is worked out to strengthen the onset and maintenance of health behavior after surgery. This part focuses on personalized outcome expectancy and self-efficacy. Temporary concomitant effects of the surgery are addressed to prepare the patients for them (treatment outcome and timeline expectations). Individual strategies are developed to deal with concomitant effects (e.g., pain or fears). In this manner, patients’ self-efficacy is strengthened. After summarizing the contents at the end of the personal session, the interventionist guides the patients to imagine their “best possible self” (59) for consolidation. The imagination expresses the success of the surgery and the recovery process by visualizing a situation with increased quality of life 3 months after surgery.

Four and eight weeks after surgery, the interventionists contact the patients in the EXPECT group via telephone for booster calls. In these calls, the interventionists and the patients talk about the implementation of activities and health behavior. Strategies that are already used are reinforced. Difficulties in implementation and solution possibilities for these are discussed. The imagination of the best possible self is repeated and refreshed. The number of booster sessions has been increased in the ValvEx trial since data of PSY-HEART I indicated that EXPECT increased personal control expectations before surgery but failed to maintain that boosted level of perceived personal control expectations after the surgery (60).

### Hypotheses

The study aims to examine if a brief preoperative psychological intervention (EXPECT) compared to standard of care (SOC) leads to a lower level of subjectively perceived illness disability 3 months after surgery. Therefore, the hypothesis μ_EXPECT_ = μ_SOC_ (μ = mean change from baseline) is tested with an α-level of 0.05 (two-sided).

### Outcome criteria

Illness-related disability 3 months after surgery is the primary outcome of the study. It is assessed with an adapted version of the Pain Disability Index (PDI) (38). For this questionnaire, representative data from the German population is available (21, 38).

Manipulation check will be assessed by a change in patients’ expectations (PDI-E, IPQ-E) (26). Secondary outcome variables are health-related quality of life [MLHFQ (39, 40), SF-12 (39)], expectations (PDI-E, IPQ-E) (26), illness beliefs (B-IPQ) (42), situational cognitive valuations (PASA) (43), preoperative anxiety level and information requirement (APAIS) (44, 45), anxiety (GAD-7) (46), depression (PHQ-9) (47), dispositional optimism (LOT-R) (48, 50), rehospitalization, length of hospital stay, length of time at intensive care unit, experience with previous surgeries, planned and realized surgery, EURO-Score II (53), STS-Score (54, 55), blood pressure, left ventricular ejection fraction (LVEF), New York Heart Association (NYHA) score (52), complications, inflammatory processes [C-reactive protein (CRP)], platelets and leukocytes and participation in rehabilitation program (e.g., cardio sport group).

### Data analysis

For examination of the primary hypothesis (H0: μ_EXPECT_ = μ_SOC_; H1: μ_EXPECT_ ≠ μ_SOC_), the illness-related disability 3 months after surgery will be analyzed using linear mixed models (baseline-adjusted, patients nested in operation type nested in centers). Main effects for group, time, and group x time will be calculated with primary focus on the difference in treatment effect (difference in mean change between baseline and 3-months follow-up between EXPECT and SOC). In doing so, a fixed significance level of α = 0.05 will be used, and Intention-to-treat-analysis (ITT) will be conducted. The reasons for upcoming missings will be analyzed, and robust maximum likelihood estimation procedures will be applied.

95% confidence intervals will be calculated for the estimation of differences in mean values. Relevant factors such as strata (center, surgery method), baseline characteristics (e.g., age, gender, level of baseline depression and anxiety score) or potential confounding (e.g., differences in interventionists) may also be considered in secondary analyses. The same procedure will be applied for secondary outcomes.

In the PSY-HEART I study, the EXPECT intervention has been shown to have partially different effects for different severities of some outcomes, for example due to the fact that the EXPECT intervention significantly decreased depressive symptoms at 6 months follow-up for patients with high baseline depressive symptoms, but not for patients with low baseline depressive symptoms (60). Therefore, covariate/moderator analyses will be conducted to examine whether the intervention is more effective for specific subgroups in terms of “what works best for whom”.

## Discussion

### Feasibility

We expect the study’s feasibility will be high as personal patient contact takes place on the day of admission. Therefore, we anticipate that recruitment goals can be met because face-to-face contact occurs only on the day of hospital admission, avoiding additional travel costs and patient journeys. The additional expenditure for patients is the time they need to complete the questionnaires and the intervention time. The booster calls are performed via telephone after 4 and 8 weeks when patients return home. Therefore, the intervention should blend in well with the hospital’s daily routine.

### Conclusion

The ValvEx-trial has the potential to reveal the effects of a brief preoperative psychological intervention on the recovery process after cardiac valve surgery. PSY-HEART I showed the positive opportunities of a preoperative psychological intervention for patients undergoing a CABG procedure (e.g., larger improvement of disability, fewer days spent in hospital) (21, 32). ValvEx is the attempt to generalize the findings to another group of patients and to examine if a shortened intervention is also capable of improving postoperative results, which was a point of debate in the PSY-HEART I trial (EXPECT-intervention_ValvEx_: approx. 70 min; EXPECT-intervention_PSY–HEART_: approx. 140 min). Until now, participation is substantially higher than in the PSY-HEART I trial, with more than three quarters of requested patients participating (77.36%). If the adapted EXPECT intervention proves to be effective, its efficacy can be verified in CABG patients and also in other surgical settings to optimize the recovery process of the widest possible range of patients.

## Ethics and dissemination

The Ethics Committees of the Department of Medicine of the Philipps University of Marburg (AZ 46/20; 2020-05-20) and the Department of Medicine of the University of Giessen (AZ 131/20; 2020-08-17) approved the study protocol, the patient information, and the informed consent (version 1, issue date: 2020-03-09). The study protocol and implementation are in conformity with the principles of the Declaration of Helsinki. The SPIRIT reporting guidelines were applied (61). All patients receive the standard medical procedure. Any protocol modifications must be confirmed by amendments in consultation with both Ethics Committees and will be communicated to investigators and participants. The written patient information and the patient consent can be found in the appendix.

In agreement with the Ethics Committees, no external data monitoring commitee and audits were necessary. Study performance was continuously monitored by the study coordinators. No interim analyses will be performed. Possible conspicuities regarding suicidality (PHQ-9) will be checked and communicated to the study centers if they occur. Additional conversations will be conducted with patients whenever there is a need. Patients will be informed that they can contact the study management with concerns at any time during and also after the study period. The study coordinators will terminate the trial when the target sample size is reached.

If patients decide to decline participation at individual measurement time points, they will still be interviewed at the other measurement time points. Similarly, patients in the EXPECT condition may decline to participate in individual conversations in the intervention. In this case, they continue participation without any changes. Any deviations from the study protocol will be recorded. Adverse events will be documented by the study investigators and will be reported to the principal investigators immediately. The principal investigators will evaluate the adverse event and advise how to deal with it. These will be reported in publications.

Only study investigators will have access to the data collected. All patient-related data will be collected with a pseudonymized subject code. Patient data will only be published in anonymized form. Data analysis and articles will be based on CONSORT criteria. Study results will be written by study coordinators and will be submitted to relevant peer-reviewed scientific journals. Study results will be presented at international congresses. Patients can ask questions about study results after study participation.

## Ethics statement

The studies involving human participants were reviewed and approved by Ethics Committee of the Department of Medicine of the Philipps University of Marburg (AZ 46/20; 2020-05-20) and Ethics Committee of the Department of Medicine of the University of Giessen (AZ 131/20; 2020-08-17). The patients/participants provided their written informed consent to participate in this study.

## Author contributions

NH: conceptualization, data curation, investigation, methodology, project administration, resources, supervision, validation, visualization, and writing—original draft. LG: conceptualization, data curation, investigation, methodology, project administration, resources, and writing—review and editing. AR: conceptualization, investigation, project administration, resources, validation, and writing—review and editing. TA, JL, MS-D, UP, MG, and SK: investigation, project administration, and writing—review and editing. AB: investigation, project administration, resources, validation, and writing—review and editing. WR: conceptualization, project administration, resources, validation, and writing—review and editing. SS: conceptualization, funding acquisition, methodology, project administration, resources, validation, and writing—review and editing. All authors contributed to the article and approved the submitted version.
